# Undernutrition and associated factors among adults living with Human Immune Deficiency Virus in Dembia District, northwest Ethiopia: an institution based cross-sectional study

**DOI:** 10.1186/s13690-016-0143-y

**Published:** 2016-07-27

**Authors:** Anbesaw Mitiku, Tadesse Awoke Ayele, Mekonen Assefa, Amare Tariku

**Affiliations:** 1 Othoniel Health Science College, Gondar, Ethiopia; 2 Department of Epidemiology and Biostatistics, Institute of Public Health, College of Medicine and Health sciences, University of Gondar, Gondar, Ethiopia; 3 Department of Public Health, College of Health sciences, Debre Tabor University, Debre Tabor, Ethiopia; 4 Department of Human Nutrition, Institute of Public Health, College of Medicine and Health sciences, University of Gondar, Gondar, Ethiopia

**Keywords:** Undernutrition, HIV positive adults, Northwest Ethiopia

## Abstract

**Background:**

Appropriate dietary intake determines the disease progression and success of Anti-Retroviral Therapy (ART). Undernutrition unacceptably increases the risk of mortality among adults living with Human Immune Deficiency Virus (HIV). However in resource limited settings including Ethiopia, many of HIV positive clients lack access to sufficient quantities of nutritious food. There is limited evidences showing the magnitude of undernutrition in this segment of the community, particularly in the rural residents. Therefore, this study aimed to assess undernutrition and associated factors among HIV positive adults attending ART clinic in Dembia District.

**Methods:**

An institution based cross-sectional study was conducted in Dembia District from October 1 to 30, 2015. Systematic random sampling technique was used to recruit the study subjects. The anthropometric measurement, Body Mass Index, was computed to determine the nutritional status of the study participants. In order to identify factors associated with undernutrition a multivariable logistic regression analysis was employed. The Adjusted Odds Ratio (AOR) with 95 % Confidence Interval (CI) was calculated to show the strength of association. In multivariable analysis, variables with a P-value of <0.05 were considered as statistically significant.

**Results:**

Of the study participants, about 23.2 % [95 % CI: 19.2, 27.2 %] were undernourished in Dembia District. The result of adjusted analysis revealed that, the odds of undernutrition was higher among adults whose age ranged between 18-29 years [AOR = 2.50, 95 % CI: 1.10, 5.69], who had a Cluster of Differentiation (CD)4 count less than 200 cells/mm^3^ [AOR = 6.21, 95 % CI: 2.97, 12.98), were widowed [AOR = 2.18, 95 % CI: 1.08,4.40), and anemic [AOR = 3.17, 95 % CI: 1.70, 5.92].

**Conclusions:**

The prevalence of undernutrition among HIV positive adults was higher in the study area. Furthermore, being in the age range of 18-29 years, widowed, anemic, and having a CD4 count of less than 200 cells/mm^3^ were positively associated with undernutrition. Therefore, efforts should be strengthened to mitigate the higher burden of undernutrition by considering the identified determinants.

## Background

Globally, 35.3 million people were living with HIV in 2012 [1]. However, there was 33 % reduction in the number of new infections. The burden remains unacceptably high in low and middle income, particularly in Africa [1, 2]. There is a complex relationship between malnutrition and Human Immune Deficiency Virus (HIV) [3]. Accordingly, HIV Infection affects nutritional status by reducing food intake and nutrient absorption, and by increasing the utilization and excretion of nutrients as the body mounts its acute phase response [3]. Because of frequent release of the pro-oxidant cytokines, HIV infection is associated with increased risk of oxidative stress causing further damage to cells, proteins and enzymes [3]. The oxidative stress and impaired immune functions mediate micronutrient deficiencies which steps-up HIV disease progression and infectivity mainly through increasing the viral load in body secretions (genital secretions and breast milk) [4].

Proper nutrition directly affects immune status, moderates the drug efficacy and side effects, and enhances the quality of life [5]. With this regard, appropriate food intake determines the disease progression and success of Anti-retroviral Therapy (ART). However in resource limited settings, many of HIV positive clients lack access to sufficient quantities of nutritious food [6, 7]. The advent of Highly Active Antiretroviral Therapy and Cotrimoxazole prophylaxis has dramatically changed the risk of morbidity, life expectancy, and course of HIV infection [3, 8, 9]. Despite tremendous advances in HIV care and funding for treatment, Acquired Immune Deficiency Syndrome (AIDS) related morbidity and mortality remains unacceptably high in developing countries [8]. Even there is significant variation in the risk of death after initiation of ART. Accordingly, the odds of death was higher among HIV positive adults with sever acute malnutrition compared to the well nourished adults [9]. Globally, more than 800 million people remain chronically undernourished, and the HIV epidemic largely overlaps with population already experiencing low diet quality and quantity [8].

The empirical evidences among HIV positive clients showed that, sex [10], residence [11], economic status [12], and educational status [13, 14] were significantly associated with undernutrition. Moreover, the presence of gastrointestinal symptoms [14, 15], opportunistic infections [14, 16], CD4count [13], eating difficulty [10, 11, 17], ART status [10], current clinical condition [17], World Health Organization (WHO) clinical AIDS stage [13, 14, 16], duration of ART [10, 17], nutritional support and dietary diversity [16], food security [13, 16, 18], and latrine availability [19] were reported as the determinants for undernutrition.

In Ethiopia since its detection, HIV/AIDS has badly costs the lives of millions. Unless the dispute of undernutrition is answered, this higher mortality rate could not be overwhelmed even in the era of ART. [10] Substantially larger number of HIV infected adults lived in the rural areas. However, the already available limited studies showing the burden of undernutrition are conducted in the referral hospitals of large cities of the country. Therefore, this study aimed to assess undernutrition and associated factors among HIV positive adults attending ART clinic in the Health Centers of Dembia District.

## Methods

### Study area and period

An institution based cross-sectional study was conducted in Dembia District from October 1 to 30, 2015. Kolladiba, the district capital, is found 729 km from the capital city of Ethiopia, Addis Ababa [20]. The district is situated at the estimated area of 1270 km^2^ and had a total population of 271,053, of which 91.4 % are rural inhabitants [21]. The livelihood of the community is largely depend on mixed farming, crop production and livestock rearing. Maize, barley, and millet are the main food crops, while rice, vetch, and chickpea are the main cash crops [20, 22]. Based on the information obtained from the District Health Office, the district has ten health centers and forty health posts. Of the total, only three health centers, Kolla Diba, Chuahit, and Aymba, has provided ART service to HIV positive clients coming from all corners of the district since 2004, 2011, and 2013, respectively. The total of 3005 HIV positive clients are registered for chronic care in the three health centers, of which 2803 were adults.

### Sample size and sampling procedure

All HIV positive adults who had at least one prior follow-up in ART clinic were eligible for the study. Using the Epi-info version-7 statistical software, a single population proportion formula was employed to calculate the sample size by considering the following assumptions: a total number (2,803) of HIV positive adults enrolled for chronic HIV care in the three health centers, 27.8 % prevalence of undernutrition [17], 95 % level of confidence, and 4 % margin of error [23]. In addition, a 10 % of non-response rate was anticipated which gives the final sample size of 453. Systematic random sampling technique was employed to select the study participants. A sampling fraction (K^th^ = N/n = 2,450/452 = 5) was calculated by dividing the monthly average number of HIV positive clients (2,450) attending ART clinic in the three health centers by the sample. The first study participant was selected using lottery method from the first five client charts at the day-one visit. Finally, the study subjects were selected at every fifth of the first client in official working days.

### Data collection instruments and procedures

A structured and interviewer-administered questionnaire was used to collect the data. The questionnaire consists of four sections: socio-demographic and economic characteristics, HIV and other medical related factors, nutritional and environmental related variables. To maintain consistency, the questionnaire was first translated from English to Amharic, the native language of the study area, and was retranslated to English by professional translators. Three clinical nurses as data collector and one public health expert as a supervisor were recruited for the study. To this effect, two days intensive training was given. The tool was piloted among twenty-three HIV positive adults attending ART clinic at Gondar health center which is out of the study area. During pre-test, the acceptability and applicability of the procedures and tools were evaluated.

Tools for measuring the dietary diversity and food security status were adopted from the Food and Nutrition Technical Assistance Project (FANTA) guidelines for measuring dietary diversity and household food insecurity access scale [24, 25]. Determination of Dietary Diversity Score (DDS) was done by asking the respondents to list all the food items consumed in the previous 24 h preceding the survey date. Then the reported food items were classified into twelve food groups. By considering the five food groups as a minimum dietary diversity [24, 26], the study participants with DDS of less than five were classified as having poor dietary diversity; otherwise, good dietary diversity if DDS was greater than or equal to five.

Food security status was assessed by using the Household Food Insecurity Access Scale. The occurrence questions used in estimating the household food security status were mainly related to the three domains: anxiety and uncertainty about the household food supply, insufficient quality (variety and preferences of the type of food), and insufficient food intake and its physical consequences. The four weeks of recall period was used, and respondents were asked to recall whether the condition in each occurrence question happened at all in the past four weeks or not. If the respondent answers “yes” to an occurrence question, a frequency of occurrence question was asked to determine whether the condition happened rarely (once or twice), sometimes (three to ten times) or often (more than ten times) in the past four weeks. Then the household food security status was defined into four categories as food secured, mildly food insecure, moderately food insecure, and severely food insecure [25].

Document/chart review was used to extract information related to HIV related characteristics. The anemia status of the study participants was ascertained using the standard criteria for female and male adults. Accordingly, if the hemoglobin level of the female study participants was less than 12.0 gm/dl, she was considered as anemic while if it was less than 13.0gm/dl for males they were defined as anemic.

The household economic status was assessed by using the selected household assets, monthly income, size of agricultural land, quantity of crop production, livestock ownership, microfinance bank account, and house ownership, and source of fuel. The principal component analysis was used to reduce 22 items to 3 (loaded as a factor 1). In the principal component analysis, the power of the variables to explain wealth status was determined step by step using the communalities values. Accordingly, variables with a communality value of greater than or equal to 0.5 were used in the final step to estimate the household wealth status. Moreover, an eigien value of greater than one was considered. Finally, the factor score was summed and ranked in to three equal categories as, highest, normal, and lowest wealth status [27].

Anthropometric measurement was carried out to determine the nutritional status of the study participants’ by using Body Mass Index (BMI). Weight of the study participants was measured using a beam balance to the nearest 0.1 Kg with a graduation of 0.1 kg and measuring range up to 160 kg. Weight was measured with lightly clothing and no shoes. Calibration was performed before weighing each participant by setting it to zero. Weighing scale also checked against a standard weight for its accuracy on daily basis. Height of the participants was measured using a seca vertical height scale standing upright in the middle of board. Participants were asked to take off their shoes, stand erect, and look straight in horizontal plain. The occipit, shoulder, buttocks, and heels touched measuring board and height was recorded to the nearest 0.01 cm [28]. Then BMI was computed by dividing weight in kilograms by the square of the height in meters (kg/m^2^). Accordingly, the study participant was defined as undernourished (underweight) if his/her BMI was less than 18.5 kg/m^2^.

### Data processing and analysis

Data were checked and entered using Epi-info version 7 statistical software and then exported to Statistical Package for Social Science (SPSS) version 20 for analysis. Descriptive statistics using cross tabulation, frequency tables, and graph were used to describe the study variables. Using binary logistic regression model, bivariable analysis was used to identify the confounders. Variables with a p-value of <0.2 were entered to multivariable logistic regression (backward stepwise selection method) to identify factors which have statistically significant association. Thus, variables having p-value of <0.05 were considered as significant. Both Crude Odds Ratio (COR) and Adjusted Odds Ratio (AOR) with 95 % Confidence Interval (CI) were used to show the strength of association. The fit of the model was assessed using the Hosmer-lemeshow goodness-of-fit test, and it was 0.58.

## Results

### Socio-demographic and economic characteristics

A total of 452 HIV positive adults were included in the study, giving a response rate of 100 %. Nearly three-fourths (71.5 %) of the respondents were females. The median (±Inter Quartile Range) age of the respondents was 35 (±12) years. Nearly two-third (59.5 %) of adults were found in the age range of 30 to 44 years. Majority of the respondents were Orthodox Christians (97.1 %), illiterate (not able to read and write) (66.2 %), and urban residents (64.4 %). More than one-third of the study participants were married (38.7 %), and divorced (38.3 %) (Table 1).

**Table 1:** Socio-demographic and economic characteristics of HIV positive adults (≥18 years) attending ART clinic in Dembia District, northwest Ethiopia, 2015

Variables	Frequency	Percent
Sex		
Male	129	28.5
Female	323	71.5
Marital status		
Single	36	8.0
Married	175	38.7
Divorced	173	38.3
Widowed	68	15.0
Religion		
Orthodox	439	97.1
Others^a^	13	2.9
Residence		
Urban	291	64.4
Rural	161	35.6
Educational status		
Cannot read and write	299	66.2
Can read and write	81	17.9
Primary education^b^	29	6.4
Secondary education^c^	30	6.6
College and above	13	2.9
Age		
18–29	83	18.4
30–44	269	59.5
≥45	100	22.1
Wealth status		
Poor	150	33.2
Medium	151	33.4
Rich	151	33.4

^a^Muslim, protestant, and catholic

^b^grade 1-8

^c^grade 9-12

### Medical related characteristics

Larger proportions (95.4 %) of HIV positive adults were fall under WHO clinical stage I, and nearly half (48.7 %) had CD_4_ count of 200-500 cells/mm^3^. Majority (96.9 %) of respondents took ART, in which most (90.0 %) of them took ART for greater than 12 months. Furthermore, about 15.5 % of adults were anemic (Table 2).

**Table 2:** Medical and dietary pattern related characteristics of HIV positive adults (≥18 years) attending ART clinic in Dembia District, northwest Ethiopia, 2015

Variables	Frequency	Percent
ART status		
Pre-ART	14	3.1
On ART	438	96.9
Duration of ART		
≤12 months	31	8.1
>12 months	407	92.9
Current CD_4_ count		
<200 mg/dl	64	14.2
200-500 mg/dl	220	48.7
>500 mg/dl	168	37.2
WHO clinical stage		
Stage I	431	95.4
Stage II and above	21	4.6
Opportunistic infections		
Yes	1	0.2
No	451	99.8
GI symptoms		
Diarrhea	1	0.2
No	451	99.8
Presence of anemia		
Yes	70	15.5
No	382	84.5
Current clinical condition		
Improved	175	38.7
Same	217	48.0
Deteriorated	60	13.3
Eating difficulty		
Loss of appetite	5	1.1
Vomiting	4	0.9
Others^a^	3	0.7
No	440	97.3
Nutritional support		
Yes	2	0.4
No	450	99.6
Dietary diversity		
Low	51	11.3
High	401	88.7
Household food security status		
Food secured	369	81.6
Mildly food secured	10	2.2
Moderately food secured	24	5.3
Severely food secured	49	10.8

^a^Swallowing difficulty and glossitis

### Prevalence of undernutrition and dietary pattern related characteristics

**Fig. 1:**
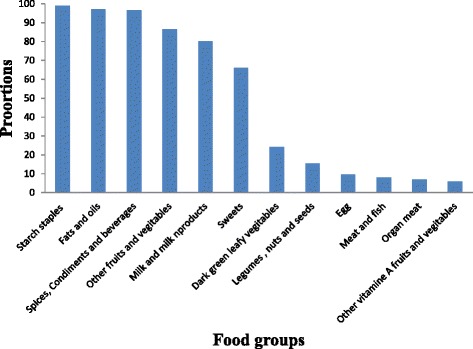
Proportion of HIV positive adults consuming individual food groups in the last 24 hours in Dembia District, northwest Ethiopia, 2015

The BMI measure taken from the respondents showed that, the mean (+SD) BMI value was 20.3 (±2.3). About 23.2 % [95 % CI: 19.2, 27.2 %] of HIV positive adults were undernourished. Only few, 0.4 %, HIV positive adults took nutritional support. Substantial proportion (88.7 %) of the respondents had high dietary diversity, and more than three quarter (81.6 %) were food secured (Table 2). Regarding the dietary pattern, the most commonly eaten food groups as stated by the respondents were starchy staples (99.1 %), fats and oils (97.1 %), and spices, condiments and beverages (96.7 %). The least food groups eaten by the respondents were other vitamin-A rich fruits and vegetables (5.8 %) and organ meat (6.9 %) (Fig. 1).

### Factors associated with undernutrition

The result of the bivariate analysis showed that, there were significant association between undernutrition and presence of anemia and a CD4 counts of less than 200 cells/mm^3^.

However, the result of the adjusted analysis showed that, the age groups of 18–29 years, marital status (widowed), presence of anemia, and having CD4 counts of less than 200 cells/mm^3^ were significantly and independently associated with undernutrition. With this regard, the odds of under nutrition was 2.5 times [AOR = 2.50, 95 % CI: 1.10, 5.69] higher among HIV positive adults whose age ranged between 18–29 years compared to those aged 45 years and above. The likelihood of undernutrition was 2 times [AOR = 2.18, 95 % CI: 1.08, 4.40] higher among widowed respondents as compared to those who were married. Moreover, the increased odds of under nutrition was noted among HIV positive adults who were anemic [AOR = 3.17, 95 % CI: 1.70, 5.92] and had CD4 count of less than 200 cells/mm^3^ [AOR = 6.21, 95 % CI: 2.97, 12.98] (Table 3).

**Table 3:** Factors associated with undernutrition among HIV positive adults (≥18 years) attending ART clinic in Dembia District, northwest Ethiopia, 2015

Variables	Under nutrition		COR (95 % CI)	AOR (95 % CI)
	Yes	No		
Age				
18–29	22	61	1.21 (0.62, 2.37)	2.50 (1.10, 5.68)*
30–44	60	209	0.96 (0.56, 1.66)	1.49 (0.78, 2.87)
≥45	23	77	1	1
Marital status				
Married	11	25	1	1
Single	39	136	0.65 (0.29, 1.44)	1.47 (0.60, 3.58)
Divorced	32	141	0.52(0.23, 1.20)	0.83 (0.46, 1.50)
Widowed	23	45	1.16 (0.49, 2.80)	2.18 (1.08, 4.40)*
Educational status				
Cannot read and write	61	238	0.85 (0.39, 1.80)	0.79 (0.34, 1.81)
Can read and write	26	55	1.56 (0.67,3.64)	1.84 (0.72, 4.66)
Primary education	8	21	1.26 (0.43,3.70)	0.81 (0.23, 2.91)
Secondary education and above	10	33	1	1
Anemia				
No	70	312	1	1
Yes	35	35	4.46 (2.61,7.61)	3.17 (1.70, 5.90)**
CD4 count (cells/mm^3^)				
<200	36	28	8.11 (4.18,15.70)	6.21 (2.97, 12.98)**
200–500	46	174	1.70 (0.97, 2.88)	1.49 (0.83, 2.68)
>500	23	145	1	1

**significant at a *p* < 0.001, *p < 0.05

## Discussion

In this study, the prevalence of undernutrition among HIV positive adults was 23.2 %. Based on the Nutrition Landscape Information System (NLIS) cut-off values, the burden of undernutrition in this study was higher [29]. This finding was consistent with other institution-based studies in Ethiopia, such as Butajira District (25.2 %) [11], Bahir Dar District (25.5 %) [10], and Gondar District (27.8 %) [17]. However, it was higher than the studies done in Singapore (16 %) [30] and Tanzania (18.4 %) [31]. This might be related to similarities in socio-economic, cultural and feeding pattern related characteristics among the study areas. The socio-cultural factors inevitably affects the clients perceived health status, response to disease and treatment outcome [32].

However, the prevalence of undernutrition was lowest compared to the study reports from other developing countries, such as Botswana (28.5 %) [15], China (37.2 %) [33], Brazil (43 %) [12], and Nigeria (43.3 %) [34]. The observed discrepancy could be attributed to the clinical stage of the study participants, where majority (95.4 %) of them found at the clinical stage one in the current study compared to the study from Brazil [12]. Obviously, late clinical stage of HIV increases the odds of developing undernutrition, mainly through higher nutritional requirement coupled with poor food intake, and malabsorption of nutrients. Moreover, the higher prevalence of undernutrition in the letter study settings could be related to sample size difference [15, 33, 34], absence of important care components, including routine nutritional screening in Botswana [15], and variations in nutritional status measurements (objectivity Vs. subjectivity) and the study design in Nigeria [34].

This study also tried to elicit the associated factors of undernutrition. Consequently, the odds of undernutrition was higher among clients whose age ranged between 18 to 29 years compared to those aged 45 years and above. The finding was unsupported by the study report from Brazil [12], in which the older aged HIV positive clients were at higher risk developing undernutrition. This is probably related to the difference in psychological makeup and life styles between the study areas. Similar to the current study area, other studies also claimed that younger adults commonly exhibited less healthy personality traits, and more vulnerable for substance abuse, and had less emotional stability to cope with stressful life events and consequences of the disease itself [35, 36]. Persistent depression attack, anxiety, and unhealthy behaviors were found to negatively affects the dietary intake and treatment outcome of the clients.

Likewise, the likelihood of undernutrition was higher among widowed study participants compared to married counter parts. This might be due to the emotion and grief encountered, and loss of spousal support. Obviously, the risk of mental illness was lower among married women than widowed [37]. Marital disruption, a stressful life event, elevates the risk of psychological distress thereby contributing to poor dietary habit and health outcomes which adversely affects their nutritional status [38].

One of the medical factors which showed a positive association with undernutrition was anemia. This finding was in line with a study conducted in Butajira [11]. This association might be due to alterations in bone marrow erythropoiesis, reduction in reticulocyte as a result of anemia [39]. In order to produce red blood cells, the bone marrow requires iron, vitamins B12 and folic acid [40]. Because of the continuing need to replenish red blood cells, the erythropoietic cells of the bone marrow are among the most rapidly growing cells of the body. Therefore, their maturation and rate of production are greatly affected by a person’s nutritional status [41]. In this respect anemia is the most commonly encountered hematologic abnormality in HIV positive patients and occurs with increased frequency as the disease progresses [40]. Bone marrow atrophy is common in anemic patients and resulted from abnormalities of stem cells or defects in stromal cells, which would alter the haemopoietic microenvironment [39]. These all interrelated facts might have a high significance in bringing undernutrition in HIV positive adults. However, the clear relationship between undernutrition and anemia necessitates further study.

In addition, the odd of undernutrition was higher among clients with CD4 counts of less than 200cells/mm^3^. The finding of this study was comparable with a study done in Nepal [13]. The CD4 is the most important hematologic indicator of how well immune system is working and the strongest predictor of HIV disease progression [30]. The progressive loss of CD4 cells eventually results in the loss of the ability to mount a desirable immune response to any pathogen resulting in death of patients in the terminal stage of Acquired Immune Deficiency Syndrome (AIDS) [42]. Weight loss has been related with impaired immune function and plays a predictive role in HIV disease progression to AIDS independently of powerful indicators, such as low CD4 cell count. Hence, the HIV-induced immune impairment and increased risk of opportunistic infections could worsen the nutritional status [30, 42].

Obviously the previous limited study reports in Ethiopia showed the magnitude of undernutrition among the urban inhabitants. However, the current study tried show the magnitude of the problem by including the rural residents. Nevertheless, some of the limitation should be noted in interpreting the study results. Firstly, hence the study was institution based, the results could not be generalized to those HIV positive adults who are not enrolled for chronic HIV care. Secondly, measurement of some of the variables, such as food intake relies on memory, as a result the study is not free from the recall bias. But, we have made efforts to reduce recall bias, including pretest, regular supervision, and training on how to probe the clients to remember their food intake.

## Conclusions

This study revealed that, the prevalence of undernutrition among HIV positive adults was higher. Furthermore, being in the age range of 18-29 years, widowed, anemic, and having a CD4 count of less than 200 cells/mm^3^ were positively associated with undernutrition. Therefore, efforts should be strengthened to provide emotional support and psychological reassurance to younger and widowed HIV positive adults. Moreover, to curve the higher burden of undernutrition, emphasis should be given to the prevention and early management of anemia and low CD4 counts by strengthening the usual hemoglobin and CD4 count evaluation.

## Abbreviations

AIDS, Acquired Immune Deficiency Syndrome; AOR, Adjusted Odds Ratio; ART, Anti-Retroviral Therapy; BMI, Body Mass Index; CD_4_, cluster of differentiation_4_; CI, Confidence Interval; COR, Crude Odds Ratio; DDS, Dietary Diversity Score; FANTA, Food and Nutrition Technical Assistance Project; HIV, Human Immune Deficiency Virus; NLIS, Nutrition Landscape Information System; SPSS, Statistical Package for Social Science; WHO, World Health Organization
